# Exploring Chinese house prices affordability in the context of confucian culture

**DOI:** 10.1371/journal.pone.0325274

**Published:** 2025-06-03

**Authors:** Fang Liu, Chen Liang

**Affiliations:** 1 Economic School, Changzhou University, Changzhou, Jiangsu, China; 2 Financial Technology School, Shenzhen University, Shenzhen, Guangdong, China; Tsinghua University, CHINA

## Abstract

Considering the notable influence of traditional Confucian culture on China’s housing market, this study introduces an innovative index to quantify the magnitude of the real estate bubble within China, employing a familial generational iterative model. Utilizing rent-buy policy as a conceptual framework, our research constructs a difference-in-differences model to investigate the impact of macroeconomic policies on the housing bubble phenomenon. Empirical observations from 2022 reveal pronounced bubble dynamics in first and second-tier cities, while housing prices in third and fourth-tier cities, alongside select fifth-tier cities, exhibit a declining trend. On a national scale, apart from minor affordability observed during 2005–2007, no significant affordability was identified in other years, with the housing price bubble index demonstrating a downward trajectory from 2020 to 2022. Furthermore, the implementation of the rent-buy policy that equality the rights of renter and owner has directly influenced the housing market, notably mitigating the overall escalation of housing prices. Additional analysis indicates that the rent-and-buy policy has been more successful in curbing price hikes in newly constructed and smaller-sized housing units compared to second-hand and larger-scale properties.

## 1 Introduction

China’s urban housing market has witnessed extraordinary price increases since the 1998 property reforms, raising serious concerns about affordability, financial stability, and social equity [1,2]. Housing prices have surged by over 570%, posing formidable challenges for aspiring homeowners and prompting widespread debate about speculative risks and the government’s role in market regulation. Local governments, heavily reliant on land sales for revenue, often implement policies that inadvertently drive-up prices [3], while the real estate sector remains a critical pillar of economic growth [4].

Against this backdrop, the rent-buy policy, introduced in 2017, aimed to ease pressure on homeownership by promoting viable rental options, especially in fast-growing urban centers with high population density [5]. Yet, understanding the policy’s effectiveness requires examining both economic and socio-cultural dimensions. Confucian norms—with strong parental support for children’s housing—reshape affordability and influence housing demand in ways that differ from Western contexts [6].

Additionally, many large cities attract a substantial non-hukou population that further amplifies housing demand. Paradoxically, this group is often underrepresented in official data—for instance, city-level statistics typically reflect only local hukou residents or formal transactions, meaning the true scope of housing demand may be underestimated. Thus, any affordability model faces the challenge of incorporating both residents (who may benefit from parental support) and non-hukou migrants (who might be more rent-oriented but still exert upward pressure on prices).

This paper therefore investigates how the rent-buy policy affects house price affordability within China’s unique socio-cultural environment. By focusing on the government’s dual role (as regulator and market participant) and cultural factors (e.g., parental financial backing), alongside the demographic complexity created by non-hukou populations, this study provides deeper insights into the persistence of high housing prices and the policy measures designed to address them. It also employs a bubble index that standardizes regional house prices relative to local income levels, thereby offering a clearer measure of affordability across diverse urban areas.

Overall, this work aims to clarify the extent of China’s housing market imbalance and evaluate potential policy pathways that could foster more sustainable and equitable housing conditions. It contributes to the broader discourse by illustrating how economic policies, cultural values, urbanization pressures, and demographic nuances intersect—ultimately shaping the successes and challenges of China’s evolving housing landscape.

## 2 Literature review

### 2.1 House price affordability

The study of economic affordability in Western academic circles gained traction in the 1970s, primarily focusing on empirical analysis and statistical methodologies. Initially, researchers concentrated on identifying and analyzing stock market affordability, with comprehensive investigations into housing market affordability emerging later in the early 21st century. Noteworthy contributions during this period include Gallin’s analysis [7], which utilized extensive time series data spanning from 1970 to 2003 to demonstrate the efficacy of the income-to-house price ratio as an indicator of housing price affordability. Similarly, Martínez-García [8] employed a dynamic panel probit framework to empirically explore the predictive capacity of macroeconomic fundamentals on episodes of exuberance in international real house prices, identifying interest rate spreads, real stock market growth, and standard housing fundamentals among the significant predictors.

Further insights were provided by Bangura [9], who observed no evidence of co-integration between real house prices and rent in the Western region, in contrast to strong evidence of co-integration in the Eastern and Northern regions. Additionally, Brzezicka [10] synthesized knowledge from various international sources on different types of price affordability, proposing a novel typology of price affordability and introducing the concept of a “conversion point,” linked to shifts in the factors driving bubble formation and changes in bubble types.

However, translating these findings to the unique market conditions of China presents challenges. The influence of China’s Confucian culture and macroeconomic policies sets it apart from Western markets, exerting distinct influences on housing prices.Liu [5] underscored the significant contribution of land prices to the escalation of house prices across China. Furthermore, Garrige [11] investigated the effects of urbanization on the housing price bubble in China, revealing a notable interrelationship between urbanization and the proliferation of housing price affordability.

In the context of Chinese society, influenced by Confucian values, the concept of housing goes beyond a mere commodity or individual asset. Housing is often viewed as a collective family responsibility, deeply intertwined with multigenerational support. This cultural expectation results in parents contributing significantly to their children’s home purchases, not only as a means of providing financial security but also to fulfill social obligations linked to family continuity and cohesion. Additionally, housing serves as a symbol of family prosperity and plays a critical role in social status, marital prospects, and intergenerational support. These cultural nuances make the Chinese housing market distinctly different from Western markets, where home purchases are generally seen as an individual endeavor.

This body of research underscores the intricate nature of housing affordability, with contributions from Western academia providing a foundational understanding of these phenomena. However, the distinct cultural and policy-driven context of China’s housing market necessitates tailored analyses to fully grasp the dynamics at play.

### 2.2 The measurement of house prices affordability

The examination of house price measurement constitutes a multifaceted endeavor intertwining economic metrics with cultural elements, particularly pertinent in the Chinese context where Confucian values exert considerable influence. This study endeavors to reevaluate the measurement of China’s house prices through the prism of social and cultural norms, aiming to offer a nuanced comprehension of the factors propelling the housing market.

Primarily, the “rental-to-sale ratio” emerges as a pivotal metric comparing a property’s monthly rent to its overall purchase price, delineated monthly [12,13]. However, gauging the perception of a house price affordability via the rental-to-sale ratio necessitates a consideration of cultural dynamics in China. Societal attitudes significantly shape the significance of homeownership, with research by Li [14] and Lin [15] indicating that owning a home is perceived as facilitating the quest for a marital partner, thus embedding property ownership as a marker of economic success and social desirability within the Chinese milieu. Moreover, the emphasis on education within Chinese families skews the housing market, with an increasing number of parents investing in properties located within esteemed school districts to secure enhanced educational prospects for their children [16]. Given these cultural nuances, the sustained demand for homeownership in China suggests that the rental-to-sale ratio may not faithfully reflect the true extent of the housing bubble. While this metric proves valuable in global contexts, its application to China’s distinct market environment may overstate the bubble’s magnitude.

Secondly, the vacancy rate, denoting the proportion of unoccupied homes relative to the total housing inventory at a given time [17], emerges as another significant metric. Methodological disparities and practical considerations in China, however, complicate the use of the vacancy rate to measure housing bubble levels accurately.

Third, the Ratio of Investment Purchases to Owner-Occupier Purchase, as proposed by Yang [18], holds promise in identifying housing market affordability to some extent. Nonetheless, challenges in acquiring precise data impede its efficacy. Turnbull [19] additionally highlights the oversight of elastic demand in this approach, where households may perceive their original homes as investments rather than residences due to enhancements in amenities.

Lastly, the house-price-to-income ratio, calculated by dividing the average house price by the average annual household income [20], offers another avenue for evaluating housing affordability. However, its reliability is contingent upon various factors, including the collective income of family members and the potential ramifications of income fluctuations on mortgage servicing capacity [21]. Liu [22] contends that this ratio provides not only a precise measurement but also rests on robust theoretical foundations.

This research accentuates the necessity for empirical studies on regulatory efficacy across diverse economic contexts and the evolution of credit markets due to technological and financial advancements. It underscores the significance of incorporating socio-cultural influences into the analysis of housing market dynamics, advocating for a comprehensive framework to comprehend housing affordability in China. The ongoing exploration of housing market measurement, encompassing both economic indices and cultural elements, stands pivotal in formulating effective policies and nurturing financial stability within a dynamic global economy.

### 2.3 Rent-buy policy

Compared to Western real estate markets, the housing sector in China has long been influenced by Confucian values, which prioritize ownership over renting. This cultural inclination has led to a slower development of the rental market in China, resulting in a structural imbalance between supply and demand. This imbalance presents challenges to achieving a sustainable and stable trajectory for China’s housing sector [23]. Furthermore, as urbanization in China progresses, the influx of workers into major cities, coupled with the entrenched belief that homeownership is essential for establishing a household, has fueled a rapid and excessive escalation in urban housing prices [24]. This trend is particularly pronounced in first – and second – tier cities (Tier 1 cities are defined as Mega cities, such as Beijing, Shanghai, Guangzhou, and Shenzhen which are major economic, cultural, and political centers with a high degree of internationalization and large populations; Tier 2 cities are Large cities, like Chengdu, Hangzhou, Nanjing, etc., which have significant economic influence and population scales within their regions), as well as regional hubs, exacerbating the affordability crisis for average families and persistently complicating efforts to address housing issues. The classification further extends to Tier 3 (Medium – sized cities), Tier 4 (Small to medium – sized cities), and Tier 5 (Small cities) as defined in the reference https://www.china-briefing.com/news/chinas-city-tier-classification-defined/.

The rent-buy policy represents an attempt to reshape the traditional emphasis on ownership and promote rental as a viable, socially accepted alternative. This comprehensive policy aims to encourage renting by providing financial incentives, enhancing tenant protections, and improving the availability and quality of rental housing.

In response, the Chinese government has implemented a dual strategy in the housing market, emphasizing both renting and purchasing. This approach aims to cultivate a more balanced housing system characterized by diverse supply sources, multifaceted support mechanisms, and integrated rental and ownership options to mitigate the surge in housing prices and address longstanding housing challenges.

From a theoretical perspective, the integration of renting and purchasing represents a significant structural reform on the supply side of the housing market [2]. This reform seeks to boost housing supply, particularly in the rental sector, to address deficiencies in rental market supply, optimize the overall housing supply structure, and foster equilibrium between rental and purchase options in the market [25]. The strategy aims to alleviate pressures on the home buying segment by nurturing a robust housing rental market, encouraging rental consumption behaviors, and diverting demand from purchasing to renting, thereby helping to mitigate the undue escalation of housing prices. Moreover, the renting and purchasing policy activates the market’s inherent self-regulatory mechanisms by offering diverse housing options, thereby enhancing the harmonious and synergistic development of the housing market.

At the core of the rent – buy policy is the expansion of rental housing availability, especially to meet the needs of “new urban citizens” (The term “new urban citizens” or “new citizens” refers to individuals whose household birth registration (hukou) and place of residence are in different cities. For example, a person might have their hukou registered in a rural or less – developed area but has moved to and is living in a more urbanized city for work or other opportunities). The objective of this policy is to promote a healthy and sustainable development of the housing market.

The Rent – Buy Policy refers to the principle of granting renters equal access to basic public services as homeowners. This concept not only ensures equal educational opportunities for the children of renters, meaning that children of renters can attend the same schools as those of homeowners in the same area without facing discrimination based on their parents’ housing status, but also extends to various public services. These services include education, eldercare (such as access to nursing homes or community – based elderly care facilities), healthcare (like being able to register with local hospitals and clinics on an equal footing with homeowners), public transportation (e.g., enjoying the same fare discounts or service quality), and other social amenities (such as access to public parks, libraries, and cultural venues).This is achieved by refining the balance of supply and demand within the rent-buy ecosystem. Unlike policies directly controlling housing prices through purchasing restrictions and financial regulations, the rent-buy approach indirectly moderates housing prices by fostering a healthy rental market and guiding rational housing consumption. This strategy underscores a long-term commitment to housing market stability and represents a cornerstone for its sustained healthy growth.

The rent-buy policy intricately balances reforms on both the supply and demand sides [26]. On the supply side, it focuses on enhancing the rental market by increasing rental housing supply and refining the rent-buy structure. On the demand side, it emphasizes guiding rational housing consumption, protecting tenants’ rights, and ensuring equality between renters and buyers. Additionally, the policy envisions creating a conducive trading environment through financial, fiscal, and tax incentives, including supportive measures for rental payments via housing funds. This comprehensive approach underscores the policy’s multifaceted commitment to facilitating a balanced, healthy, and dynamic housing market.

The rent-buy policy was implemented in 2017 as part of a broader effort by the Chinese government to address rising housing prices and improve the affordability of rental housing. Initially, the policy was introduced in several major cities as a pilot program, targeting urban areas with high population density and rapidly escalating housing prices. The main objective of the policy was to create a more balanced housing market by promoting rental housing as a viable alternative to homeownership.

Given the structural changes initiated by the rent-buy policy, it is important to evaluate its impact on housing prices in both the short and long term, as analyzed in the following section.

### 2.4 Rent-buy policy and house price

Analyzing the theoretical foundations of the rent-buy policy underscores its considerable potential to influence housing prices in both the short and long terms within the context of the housing market. In the short term, this strategy impacts housing supply and alters public expectations regarding homeownership. Meanwhile, in the long term, it reshapes the supply-demand dynamics within the housing market, consequently affecting housing prices.

In the short term, the rent-buy policy conveys a clear message about the trajectory of housing market development by emphasizing the expansion of the housing rental market and augmenting rental housing supply [27]. This approach suggests that housing needs can be effectively met through rentals, thereby altering public expectations regarding perpetual price increases [28]. Consequently, it steers consumer behavior towards more rational decisions when deciding between renting and buying, thereby moving away from panic-driven purchases and potentially mitigating the rapid escalation of housing prices.

Over the long term, the rent-buy policy fosters the rapid development of the rental market, redirecting some demand from the crowded housing market. This alleviates competitive pressures and mitigates the driving forces behind rising housing prices [29]. Moreover, the implementation of the rent-buy policy, alongside subsequent policies ensuring equal rights for renters and buyers, breaks down barriers between the housing and rental markets. This facilitates a more fluid transition between renting and buying, efficiently catering to both the essential needs of new citizens and the desires of existing residents for housing improvement. The policy fosters an integrated and synergistic development within the housing market, contributing to the suppression of upward trends in housing prices. Thus, this paper posits Hypothesis 1:

Hypothesis 1: The rent-purchase policy exerts a moderating effect on housing prices, irrespective of the time frame considered.

The rent-buy policy adeptly adjusts the structure of the housing market by fostering the growth of the rental market and balancing supply-demand dynamics through the augmentation of new housing and the revitalization of existing stock. Molloy [30] suggests that the housing market encompasses both new housing and secondary markets, with new constructions contributing to the increment of housing stock and used homes constituting the existing stock. Byrne [31] indicates that the rent-buy policy does not directly impact the secondary housing market, as the supply of used homes remains relatively stable in the short term. However, the supply of new constructions can swiftly adapt in response to policy shifts, indicating higher sensitivity to regulatory changes and a more pronounced policy impact.

Regarding market demand, the rent-buy policy accelerates the integration of the rental and home-buying markets, facilitating a smoother transition between renting and purchasing. Given the inverse relationship between housing prices and the size of living space, the most sensitive market segments to these changes include new citizens and lower-income groups. These individuals are likely to make the most rational housing choices, navigating between renting and buying, as well as between larger and smaller living spaces, based on the unfolding of the rent-buy policy. Consequently, this paper posits Hypothesis 2:

Hypothesis 2: The rent-buy approach will adjust housing demand, exerting varied impacts across different housing types.

### 2.5 Migration and housing policy

Housing policies, particularly those targeting affordability, can have significant effects on population migration patterns. In China, migration is often influenced by the availability and affordability of housing, as individuals move to areas with better economic opportunities and more accessible living conditions [32]. The rent-buy policy, aimed at improving the affordability of rental housing, may alter migration flows by making certain cities more attractive to potential residents [3].

The availability of affordable rental housing can encourage migration, especially for younger individuals or families who might otherwise be deterred by high homeownership costs. By enhancing the rental market and providing more housing options, the rent-buy policy could potentially increase the influx of migrants to treated cities, particularly those seeking employment opportunities in urban areas [33]. Conversely, improved rental conditions may also lead to a reduction in out-migration from these cities, as residents are able to find affordable housing options without needing to relocate [34].

Hypothesis 3: The rent-buy policy has a significant impact on population migration, potentially affecting migration into or out of the treated cities.

### 2.6 Research gap and contribution

In summary, Western-based affordability metrics and general housing theory offer valuable starting points for understanding house price determinants. However, key gaps remain in the context of China’s unique market, where:

Confucian culture fosters intergenerational financial support, influencing both affordability metrics and demand for homeownership.Government-led strategies, like the rent-buy policy, seek to reshape entrenched cultural norms and structural imbalances but have not been examined extensively in empirical research that directly measures policy impacts on new versus second-hand housing markets.Demographic factors, including non-hukou populations and internal migration, can amplify or dampen policy effects yet are often excluded in existing affordability models or official data.By addressing these dimensions, the present study bridges the theoretical and empirical gaps. It applies a multifaceted approach that integrates cultural, demographic, and policy-specific variables into the assessment of housing market dynamics in China. The next section outlines the methodological framework employed to capture these complexities and evaluate the rent-buy policy’s efficacy in moderating house price escalation.

## 3 Model and data sources

### 3.1 The index of house price affordability model

To clarify, “House Price Affordability” in this study refers to the maximum house price a household can reasonably afford without compromising their overall quality of life, but with particular emphasis on China’s traditional cultural norms. Unlike the more individual-oriented affordability metrics commonly employed elsewhere [35], this research recognizes that Confucian values strongly influence Chinese society by reinforcing intergenerational bonds and extended financial support within families [36]. Consequently, adult children in urban China often receive considerable monetary assistance from their parents when purchasing a home, which expands both the total wealth available for a down payment and the income base considered during mortgage repayment [37].

In this context, the affordability ceiling for home purchases depends not only on the buyer’s earnings but also on the family’s overall wealth and projected disposable income, incorporating cultural norms that place paramount importance on homeownership as a marker of family stability and social status [38]. This family-centric view of affordability naturally differs from simpler, individual-based metrics and offers a more holistic lens through which to examine China’s housing market.

Building on these cultural-economic realities, the model developed in this study includes socio-cultural indicators reflective of Chinese norms, integrates the possibility of household private loans, and accounts for two-generation joint income. By approaching affordability from this broader perspective, the research captures how parental involvement—both financially and socially—can raise the affordability threshold, while at the same time highlighting the unique features of China’s housing sector that may not be as relevant in Western contexts. Although such an approach inevitably omits fine-grained household-level income distribution details, existing literature suggests that house price fluctuations are still strongly correlated with overall affordability at the macro level [39,40]. Therefore, despite potential limitations, this culturally nuanced, family-centered framework provides a more accurate depiction of how traditional Chinese culture shapes housing affordability, reflecting real-world dynamics in which intergenerational support plays a defining role.

#### 3.1.1 Static model of house price affordability.

The foundational structure of our model draws parallels to the framework established by Lv [41], focusing on the dynamics of house pricing. Within this model, ρ denotes the price of a house, while ‘n’ represents the proportion of the down payment relative to the house’s total cost. ϑ is identified as the monthly mortgage payment, and γ signifies the monthly interest rate applicable to the mortgage loan. The duration of the bank loan, expressed in months, is captured by τ. Additionally, ∈ delineates the fraction of urban households’ monthly disposable income that is allocated towards mortgage payments. The term λ is defined as the ratio of a family’s total consumption expenditure to its total disposable income, providing insight into spending patterns.

Price = the sum of the down payment + monthly discounted value, i.e.,:


$$\begin{array}{c} P=\eta P+\frac{\vartheta }{(1+r{)}^{1}}+\frac{\vartheta }{(1+r{)}^{2}}+\frac{\vartheta }{(1+r{)}^{k}}\cdots \cdots \frac{\vartheta }{(1+r{)}^{\tau }}=\eta P+\frac{\vartheta }{r}\frac{(1+r{)}^{\tau }-1}{(1+r{)}^{\tau }} \end{array}$$ (3.1)

Assume that the monthly disposable income of urban households excluding consumption expenditures, is used to make the monthly payments.


$$\begin{array}{c} \vartheta =\frac{Y\in }{12}=\frac{Y(1-\rho )}{12} \end{array}$$ (3.2)

Substituting (2) into (1) gives:


$$\begin{array}{c} P=\frac{Y(1-\lambda )}{12r(1-\eta )}\left(\frac{(1+r{)}^{\tau }-1}{(1+r{)}^{\tau }}\right) \end{array}$$ (3.3)

Equation (3) theoretically determines the reasonable upper limit of the total house price that urban residents can afford.

Set $P_{\max}=\frac{P}{e}$, to facilitate comparison of different regions of the housing affordability, we define the bubble index as follows:


$$\begin{array}{c} S_{1}=\frac{P_{maket}}{P_{\max}}=\frac{P_{market}}{\frac{Y(1-\rho )}{12r(1-\eta )}\left(\frac{(1+r{)}^{\tau }-1}{(1+r{)}^{\tau }}\right)} \end{array}$$ (3.4)

$P_{market}$ is the average price per square metre, and $P_{\max}$ is the upper limit price per square meter. If S > 1, then there is a house price affordability. Otherwise, the bubble does not exist. Where e represents the average household income in the region. This variable is used to normalize the house price P by the income level, which facilitates comparison across different regions with varying income levels. By defining e as the income factor, we ensure that the bubble index reflects the affordability of housing relative to the local economic context.

#### 3.1.2 Family’s composition and income structure dynamic model.

Model 1 assesses the maximum housing prices society can withstand from a static viewpoint. Nonetheless, to preemptively mitigate financial risks, it’s imperative to adopt a dynamic approach that accounts for the potential for default throughout the loan term. Considering that mortgage durations often extend between 20–30 years, significant changes in a family’s composition and income structure are likely within such a timeframe. Thus, a realistically sustainable upper price limit should fall below that derived from Model 1. For the sake of managing financial risk, the maximum monthly payment calculation in equation (3.2) should not consider the income contribution from the oldest family members.

Inspired by the overlapping model of Diamond [42], we assume that there are three generations in a society. The average number of children per couple in the first generation is  ω, and the average number of children per couple in the second generation is ε, the ratio of the first, second and third generation population in the total population of the society is 2: ω: ε, that is, the ratio of three generations is: $\frac{4}{4+2\omega +\varepsilon }:\;\frac{2\omega }{4+2\omega +\varepsilon }:\frac{\varepsilon }{4+2\omega +\varepsilon }$. Subtract the first generation’s contribution, and equation (3.3) becomes:


$$\begin{array}{c} P=\frac{2\omega +\varepsilon }{4+2\omega +\varepsilon (1-\eta )}\frac{Y(1-\rho )}{12r(1-\eta )}\left(\frac{(1+r{)}^{T}-1}{(1+r{)}^{T}}\right) \end{array}$$ (3.5)

Correspondingly, equation (3.4) is modified to:


$$\begin{array}{c} S_{2}=\frac{P_{maket}}{P_{\max}}=\frac{P_{maket}}{\frac{2\omega +\varepsilon }{4+2\omega +\varepsilon (1-\eta )}\frac{Y(1-\rho )}{12r(1-\eta )}\left(\frac{(1+r{)}^{T}-1}{(1+r{)}^{T}}\right)} \end{array}$$ (3.6)

The above models only consider the case of fixed income, but in fact, per capita income increases every year, which means that the highest affordable price is increasing over time. We assume that the average monthly income growth is g, and equation (3.2) can be modified to:


$$\begin{array}{c} D_{k}=\frac{Y(1+g{)}^{k}m}{12}=\frac{Y(1-\rho )(1+g{)}^{k}}{12} \end{array}$$ (3.7)

where k is the kth repayment period, k = 1...T. Substituting equation 3.7 into equation 3.1, and then considering the factors included in Model 2, we get:


$$\begin{array}{c} S_{3}=\frac{P_{market}}{P_{\max}}=\frac{P_{market}}{\frac{2\omega +\varepsilon }{4+2\omega +\varepsilon (1-\eta )}\frac{Y(1-\rho )}{12r(1-\eta )}\left(\frac{(1+r{)}^{T}-1}{(1+r{)}^{T}}\right)} \end{array}$$ (3.8)

Comparing equation (3.8) with equation (3.6), we find


$$S_{3}=\frac{r-g}{r}S_{2}$$ (3.9)

We call the coefficient $\frac{r-g}{r}$ the bubble absorption factor. In other words, due to the increase in income over time, the previously formed bubble will be automatically” absorbed”, which is like the self-healing function of the human body. For example, if a healthy person, through staying up late develops acne, with a proper work and rest balance, the acne will be automatically absorbed by the body without the need for medication or surgery. This phenomenon provides an idea for resolving the current housing bubble problem. Monetary policy can be used to adjust the interest rate of loans, and fiscal policy can affect the disposable income of residents. If the monetary policy and fiscal policy are used together properly to support a healthy economy, then people’s income should increase steadily, and at the same time house prices can be made to rise more slowly than the increase in income. The bubble will be automatically absorbed without posing a threat to the financial system. It is worth noting, however, that if the macro-control measures are used improperly, the economy will decline, the income of residents’ decrease, and in this case, the bubble absorbing factor may instead become a bubble amplification factor.

### 3.2 Basic model

The collaborative pilot reform of the rent-and-purchase housing system—initiated by the Ministry of Housing and Urban-Rural Development in conjunction with nine other ministries—provides a unique policy experiment for assessing how rent-purchase and rent-elevation measures influence house price affordability. Given this quasi-experimental setting, we adopt the Tan [43] model and apply a Difference-in-Differences (DID) methodology to rigorously estimate policy impacts.

The DID approach is particularly well-suited to policy evaluations of this nature because it compares changes in housing outcomes before and after the reform in treatment (pilot) cities to changes in control (non-pilot) cities over the same period. By using these two sets of cities and tracking them longitudinally, DID helps isolate the policy effect from other time-varying economic or social factors that could simultaneously affect house price affordability. This ensures that any observed differences can be more confidently attributed to the rent-purchase policy rather than to broader macroeconomic shifts or city-specific trends.

Drawing on Tan [43], we specify the following model to capture the policy’s average treatment effect on house price affordability:


$${HPA}_{it}=\alpha_{0}+\alpha_{1}{\text{ Treat }}_{it}\times {\text{ Policy }}_{it}+\sum_{k}\;\beta_{k}x_{it}^{k}+\eta_{i}+\lambda_{t}+\varepsilon_{it}$$ (3.10)

In equation (3.10), where ${HPA}_{it}$ denotes the house price affordability of the icity in periodt; ${\;Treat}_{it}$ denotes the dummy variable for whether the icity is a pilot city in in periodt, which takes the value of 1 if it is, and 0 otherwise; and ${\text{ Policy }}_{it}$ denotes the experimental staging dummy variable for whether or not to implement a rent-and-buy policy, which takes the values of 0 and 1.${\text{ Treat }}_{it}\times {\text{ Policy }}_{it}$ is the interaction term between the experimental grouping and the experimental staging dummy variable, whose coefficient indicates the net effect of implementing the rent-and-buy policy; $x_{it}^{k}$ is a series of other control variables mentioned above; $\eta_{i}+\lambda_{t}$ is denotes the individual city fixed effect, and the time fixed effect, respectively. and $\varepsilon_{it}$ is a random disturbance term.

Based on the principle of the double-difference approach, this paper focuses on the coefficient 1 of the interaction term${\text{ Treat }}_{it}\times {\text{ Policy }}_{it}$, which represents the net effect of the rent-and-buy policy on housing price affordability after excluding other disturbances. If 1 is less than zero, it indicates that the rent-and-buy policy improves housing price affordability, and vice versa, it worsens housing price affordability.

This structure leverages the parallel trends assumption—that, in the absence of the policy, the trajectories of the treatment and control groups would have evolved similarly. Given the pilot nature of the reform and the availability of pre- and post-policy data, DID becomes a robust tool to estimate how the rent-purchase policy directly affects house price affordability. By contrasting the differential shifts across these groups, we can more confidently deduce whether the reform indeed moderated housing costs and improved affordability in the target cities.

### 3.3 Data source

This study examines the impact of the rent-purchase policy on housing prices across 130 large and medium-sized Chinese cities. It specifically focuses on 166 cities involved in the rent-purchase pilot program as the experimental group, contrasting them with the remaining 296 cities designated as the control group. The objective is to assess the policy’s influence on both new and second-hand house prices over a significant period, from 1987 to 2022, utilizing extensive panel data.

The research draws on the 2017 joint directive titled “Notice on Accelerating the Development of the Housing Rental Market in Large and Medium-sized Cities with Net Population Inflows” issued by the Ministry of Housing and Urban-Rural Development and nine other ministries. This directive serves as a key reference point in understanding the context and objectives of the rent-purchase policy.

To comprehensively evaluate the policy’s effects, the study incorporates a range of control variables known to impact housing price dynamics. These variables include population size, the number of elementary school students, per capita disposable income, tertiary industry value-added, GDP, rental prices, housing supply, commercial residential property sales, new house numbers, and second-hand house prices. Additionally, the analysis considers factors such as commercial residential sales, investment, and land prices.

Data for the study is sourced from reputable databases and reports. The Wind Information database provides essential information on housing prices, rents, residential land prices, sales area, and investment figures. The China Housing Price Quotation Network supplies data on rental housing supply, while demographic and economic indicators are obtained from the Wind Data Center, recent China Urban Statistical Yearbooks, and municipal statistical reports.

By leveraging this comprehensive dataset and analytical framework, the study aims to provide valuable insights into the rent-purchase policy’s impact on housing price dynamics in Chinese cities, offering policymakers and stakeholders a better understanding of its implications and effectiveness.

## 4 The measurement of house price and variables

### 4.1 The measurement of house price

Utilizing both static and dynamic models to analyze housing prices, the findings reveal a substantial disparity in identifying housing affordability. The static model (Model 1) identifies 40 cities with housing affordability, while the more comprehensive dynamic model (Model 2) uncovers 126 cities grappling with this issue. This variance underscores the significant influence of familial and cultural factors on housing prices in China.

According to Model 1, 40 cities exhibit a bubble index exceeding 1, indicating considerable overvaluation. Furthermore, 72 cities display a bubble index ranging from 0.8 to 1, with the remaining 223 cities falling below the 0.8 threshold. Housing affordability are predominantly concentrated in the Eastern regions, occasionally extending to Central and Western cities.

Model 2 expands on these findings, identifying an additional 86 cities experiencing long-term housing affordability, thereby highlighting underlying financial risks. Within this model, 20 cities approach the bubble threshold, with indices between 0.9 and 1.0, indicating the potential for widespread financial instability with slight changes in parameter estimates. This model reveals a broader distribution of housing affordability, with 126 cities having indices above 1, signifying significant overvaluation across both Eastern and Midwest regions of China. Moreover, 90 cities have affordability indices between 0.8 and 1, and 128 cities have indices below 0.8. This comprehensive assessment points to considerable affordability across a wider geographic spectrum.

It is worth noting that Model 2 is a dynamic model that integrates the income growth of various household economies, influenced by the economic growth of each region. Due to the diverse economic conditions across these regions, predicting the average income growth (referred to as Ave growth in the Table 1) of the 296 municipalities over the next 29 years poses a significant challenge for this study.

**Table 1:** China^’^s economic fundamentals 1987-2022.

Year	Ave growth	Mortgage rate	Engel coefficient	Aging rate	Absorption rate
1987	13.10%	10.08%	53.50%	9.90%	0.75
1988	13.08%	13.32%	51.40%	10.24%	0.70
1989	12.98%	19.26%	54.50%	10.59%	0.63
1990	12.93%	11.52%	54.20%	10.94%	0.72
1991	12.86%	10.80%	53.80%	11.28%	0.73
1992	12.61%	9.72%	53.00%	11.60%	0.74
1993	11.93%	13.14%	50.30%	11.86%	0.67
1994	10.75%	14.04%	50.00%	12.08%	0.61
1995	10.01%	15.03%	50.10%	12.28%	0.57
1996	9.77%	13.77%	48.80%	12.45%	0.58
1997	9.82%	10.53%	46.60%	12.60%	0.62
1998	9.98%	8.64%	44.70%	12.84%	0.65
1999	9.98%	6.21%	42.10%	13.04%	0.69
2000	9.76%	6.21%	39.40%	13.30%	0.68
2001	9.44%	6.21%	38.20%	13.59%	0.66
2002	8.96%	5.76%	37.70%	13.88%	0.65
2003	8.61%	5.76%	37.10%	14.31%	0.63
2004	8.19%	6.12%	37.70%	14.72%	0.60
2005	7.77%	6.12%	36.70%	15.01%	0.58
2006	7.32%	6.62%	35.80%	15.57%	0.55
2007	6.62%	7.49%	36.30%	16.24%	0.50
2008	6.06%	6.89%	37.90%	17.02%	0.48
2009	5.77%	5.94%	36.50%	17.89%	0.47
2010	5.36%	6.27%	35.70%	18.86%	0.44
2011	4.82%	6.82%	36.30%	19.89%	0.40
2012	4.36%	6.68%	36.23%	20.96%	0.37
2013	4.03%	6.55%	35.00%	22.05%	0.35
2014	3.83%	6.15%	34.20%	23.14%	0.34
2015	3.58%	5.40%	29.70%	24.20%	0.32
2016	3.34%	4.90%	29.30%	25.22%	0.31
2017	3.08%	4.90%	28.60%	26.19%	0.29
2018	2.85%	4.90%	27.70%	27.10%	0.27
2019	2.71%	4.76%	27.12%	27.57%	0.24
2020	2.68%	4.61%	26.51%	27.22%	0.21
2021	2.47%	4.51%	26.13%	28.31%	0.19
2022	2.19%	4.47%	25.95%	28.19%	0.15

It can be seen from Table 1 that before the mid-1990s, because the proportion of older generation contributors was low, only 10%, the results of Model 1 and Model 2 did not differ much. As the proportion of the older generation increases, the gap between Model 1 and Model 2 becomes wider, reaching 28.19% in 2022.

Before 2000, the bubble absorption rate, calculated as 1 minus the absorption factor representing the proportion of the “bubble” not being absorbed, was very high, reaching a maximum of 75% in 1987.Although there were some fluctuations, the economic system could absorb more than 60% of the bubble on average. After 2000, the absorption rate decreased year by year, from 68% in 2000 to 28.19% in 2022. The high absorption rate before 2000 was mainly attributable to two reasons. Firstly, in the 1990s, the proportion of the aging population was small (about 10%), the supply of young and middle-aged labor was abundant, the economic was developing fast, and the income growth rate was high. Secondly, the decline in mortgage rates during this period was relatively large (from 19% down to 6%) and the interest burden was therefore reduced significantly. For the above reasons, the economic system played a significant part in absorbing the bubble. This shows that in a society with a strong economy and loose monetary policy, the short-term property bubble will be absorbed quickly. After 2000, as China gradually entered the ranks of moderately developed countries, income growth gradually slowed down, and there was little room left for interest rate to decrease (4.9% in 2016). Also, the percentage of the population in the older age bracket soared (28.19% in 2022), making the economic system less able to absorb housing affordability.

This analysis offers fresh perspectives on managing the housing price bubble. A feasible approach involves adopting a more lenient monetary policy to reduce mortgage interest rates without adversely affecting other economic sectors. Concurrently, adopting proactive fiscal policies, such as tax reductions to boost disposable incomes, could markedly mitigate the bubble’s economic impact. Long-term solutions to the housing bubble issue hinge on ensuring sustained, stable economic growth, whereby residents’ disposable incomes grow steadily, contributions from extended family remain viable, and macroeconomic policies are adeptly applied, ensuring the real estate market’s stability and prosperity.

### 4.2 Variables description

**Dependent Variable:** This study initially posits that Confucian cultural norm particularly intergenerational financial support and a strong emphasis on home ownership significantly shape housing affordability. While an ideal empirical approach would incorporate direct affordability metrics (e.g., price-to-income ratios), consistent household-level data spanning 1987–2022 are unavailable. Consequently, we use house prices as a practical proxy for affordability, reflecting the premise that higher prices generally impose greater financial burdens [35]. Although this proxy does not fully capture all dimensions of affordability, it aligns with the broader theoretical context by indicating how cultural values drive demand—and thus prices—in the Chinese housing market.

Moreover, we recognize concerns regarding the accuracy of house price data from Sofun.com, including the possibility of listed prices differing from final transaction amounts. To mitigate such issues, we performed cross-validations with alternative sources, notably Lianjia.com and local government records in selected major cities, observing consistent patterns in aggregated price trends despite occasional micro-level discrepancies. Additionally, a data cleaning procedure removed evident outliers or incomplete listings, and key regressions were tested on a subsample cross-verified with Lianjia.com to confirm the robustness of the findings. Although using a single commercial platform entails potential measurement error, Sofun.com remains one of the most extensive longitudinal data repositories for the period under study. By acknowledging these data constraints, undertaking validation checks, and aligning our measure of affordability with the cultural framework posited, we endeavor to reconcile the theoretical emphasis on Confucianism with the practical realities of empirical modeling. Future research may enhance this link by incorporating more granular affordability indicators—such as household income distributions—while still accounting for cultural norms that influence housing decisions in China’s unique market context.

**Independent Variable**: Policy Intervention. This analysis references the “Notice on Accelerating the Development of the Housing Rental Market in Large and Medium-sized Cities with Net Population Inflows,” issued in July 2017 by the Ministry of Housing and Urban-Rural Development and nine other ministries. The data are segmented into experimental and reference groups based on the policy’s introduction.

**Control Variables:** Include rent price, population, elementary school enrollment figures, per capita disposable income, value-added of the tertiary sector, gross domestic product (GDP), and the supply of rental housing. Data on rent and land prices are acquired from the Wind Data Center. Information regarding the supply of rental housing is derived from the China Housing Price Quotation Network. Additionally, data on resident population, elementary school student numbers, per capita disposable income, tertiary industry value-added, and GDP are collated from the Wind Data Center, the China Urban Statistical Yearbook, and municipal statistical reports.

Examination of Table 2 reveals significant disparities in regional house price affordability across China, highlighting a concentration of affordability in first and second-tier cities. Furthermore, an analysis of the control variables indicates substantial regional differences in education levels, tertiary sector output, and GDP per capita. This underscores the prevalent regional imbalances in China’s development.

**Table 2:** Variables description.

Variables		Min	Max	Mean	S. D	Obs
Dependent variables	House price affordability	0.561	1.712	1.07	1.02	6975
Independent variables	Rent-buy policy	0	1	0.27	0.037	6975
Control variables	Population	9.796	37.212	13.411	12.765	6975
	Elementary school	3.471	10.65	4.371	1.95	6975
	Disposable income(ln)	0.971	12.371	1.272	0.972	6975
	Tertiary industry	0.712	4.563	1.021	0.872	6975
	Per GDP	7.78	11.21	10.041	0.691	6975
	Rental house price	0.792	11.271	4.357	0.382	6975
	Land price	2.312	8.732	3.212	1.271	6975
	Suburban housing supply	0.212	0.981	0.571	0.712	6975

## 5 The impact of rent-buy policy on real estate industry

### 5.1 Rent-buy policy

After over four decades of reform, the urban housing system in China has undergone profound transformations, evolving through stages that include welfare provision, commercialization, property rights establishment, and financialization. Concurrently, regulatory frameworks overseeing the housing market have progressed from comparative analyses of renting-selling and renting-buying to the current paradigm, which prioritizes ensuring parity between renting and buying rights.

Initially conceived in the 1990s, the “joint development of renting and selling” policy aimed to bolster housing commercialization by augmenting supply. However, contemporary policies such as the “joint development of renting and buying” and “parity of rights between renting and buying” prioritize addressing housing demands through supply augmentation.

Under these policy frameworks, there is a dual emphasis: stimulating market development through increased purchasing activity while concurrently fostering growth in the rental sector. Safeguarding equitable rights for both tenants and buyers assumes paramount importance, not only to protect homeowners’ interests but also to facilitate equitable access to public services.

In the preceding era, the Chinese government enacted an array of welfare-oriented policies within the housing domain to ensure populace access to housing, exemplified by initiatives like subsidized housing and public rental schemes. However, in consonance with temporal progression, concurrent with the evolution of market dynamics and the paradigm of economic liberalization, the trajectory of China’s housing market has undergone a discernible shift toward financialization. This paradigmatic shift denotes an augmented influence of financial capital and market mechanisms within the housing sector, encompassing activities such as real estate investment, mortgage financing, and the proliferation of financial derivatives. This transformation has precipitated a persistent uptrend in real estate valuations. Correspondingly, regulatory emphasis has transitioned from the antecedent paradigm of contrasting rental and ownership arrangements to the contemporary emphasis on juxtaposing rental and ownership modalities, undertaken with the overarching aim of more effectively addressing fundamental societal imperatives pertaining to habitation.

Theoretically, these reforms target the supply side of the housing market. By increasing housing supply, particularly in leasing, shortages can be addressed, and the balance between leasing and purchasing dynamics can be achieved. This approach also eases pressure on the housing market, thereby curbing excessive price increases and promoting a harmonious and healthy market.

The focus of rent-buy policy lies in augmenting rental housing supply, particularly for new citizens, to achieve a sustainable housing market. Notably, this policy diverges fundamentally from direct price regulation measures like purchase restrictions, focusing instead on cultivating a robust rental market to guide rational housing consumption, ultimately fostering a healthy market development.

Theoretical implications suggest that rent-buy policies significantly impact housing prices both short and long-term. In the short term, they influence supply and alter buying expectations, thereby rationalizing consumer choices and mitigating price surges. In the long term, such policies alleviate market competition by diverting demand to rentals, eroding upward price pressures. Additionally, they break down barriers between rental and ownership markets, facilitating smoother transitions and providing diverse housing options for both new and existing residents, ultimately tempering price escalation.

Hence, it is proposed herein that rent-buy policies exert a moderating influence on housing prices, both in the short and long term.

### 5.2 The effect of rent-buy policy on house price affordability

This section compares new (first-hand) house prices and second-hand house prices between the treatment and control cities before and after the enactment of the rent-buy policy (see Table 3). Columns (1) report the main Difference-in-Differences (DID) results using Sofun.com data, whereas columns (2) replicate the analysis using Lianjia.com data as a robustness check. The policy effect, captured by the “Treat × Policy” coefficient, remains negative and statistically significant in both data sources, indicating that the rent-buy policy effectively dampened house price growth—hence improving affordability. Specifically, the estimates in columns (1) suggest a reduction of about 5.1% in new house prices and 7.1% in second-hand house prices; while columns (2) using Lianjia.com data yield smaller but still significant effects of 2.7% and 1.5%, respectively. These consistent findings help alleviate concerns regarding data accuracy, affirming that the main results are robust across different platforms.

**Table 3:** Difference in difference.

Variables	First hands house price (1)	Second hands house price (1)	First hands house price (2)	Second hands house price (2)
Treat*policy	−0.047*** (0.012)	−0.069*** (0.013)	−0.027** (0.012)	−0.015* (0.009)
Control variables	Y	Y	Y	Y
Fix effects	Y	Y	Y	Y
Time effects	Y	Y	Y	Y
R	0.417	0.472	0.129	0.217
Obs	6975	6975	3121	3121

***, **, and * indicate 1%, 5%, and 10% significance levels, respectively.

From a Confucian cultural perspective, this policy aligns with traditional values emphasizing social harmony, familial stability, and economic well-being. Confucianism advocates for creating a balanced and fair society, where the needs of the family and community are prioritized. The rent-buy policy, by addressing the rising costs of housing, contributes to reducing social inequalities and enhancing access to homeownership, which resonates with the Confucian ideal of social equity and harmony.

**Fig 1:**
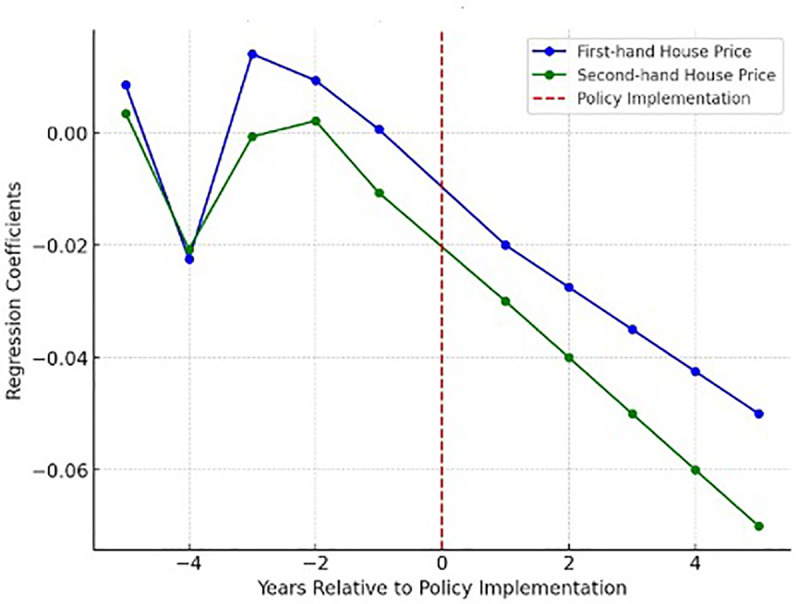
Trend test.

To confirm the validity of the DID approach, it is crucial that the parallel trends assumption hold—that is, house price trajectories in treatment and control cities would have evolved similarly in the absence of the policy. Fig 1 presents the common trend test, showing that none of the regression coefficients for the years prior to the policy reach the 5% significance level. This implies no statistically significant pre-policy divergence in price trends, lending support to the parallel trends assumption.

Moreover, the selection of treatment and control cities was guided by their similarity in key economic indicators before the policy intervention, including baseline house prices, average household income levels, economic growth rates, urbanization rates, and population density. This matching procedure further enhances the credibility of the parallel trends assumption by ensuring that, absent the policy, both groups of cities would likely have experienced comparable house price trajectories. This matching process also reflects the Confucian cultural emphasis on maintaining a sense of balance and equity across regions, as the policy aimed to ensure that the treatment and control cities were similar in terms of their economic contexts.

Overall, the DID estimates demonstrate that the rent-buy policy has a measurable, negative effect on both new and second-hand house prices, thus improving house price affordability—a core concern of this study’s focus on Confucian cultural contexts. The application of Confucian principles in policy design has shown potential in promoting social equity and well-being, key values within Confucianism. Equally important, the robustness check using Lianjia.com data supports the reliability of the Sofun.com-based findings, underscoring that concerns over listing accuracy do not materially alter the conclusion that the policy has curbed house price growth.

To visualize the results of the common trend test and the impact of the rent-buy policy on house prices, the test results are plotted in Fig 1. As shown in the figure, the regression coefficients for first-hand and second-hand house prices before the policy are close to zero, with no statistically significant deviations, which suggests that the treatment and control cities had similar trends prior to the policy. This supports the parallel trends assumption, which is critical for the validity of the Difference-in-Differences (DID) estimation employed in this study.

After the policy implementation, the regression coefficients exhibit a significant negative trend for both first-hand and second-hand house prices, consistent with the findings in Table 3. Specifically, the policy effect is estimated as −0.051 for first-hand house prices and −0.071 for second-hand house prices, both statistically significant at the 1% level. This downward trend post-policy indicates that the rent-buy policy effectively reduced house price growth over time.

While the pre-policy trends in Fig 1 suggest no significant difference between the treatment and control cities, it is important to note that the observed negative coefficients in Table 3 are a result of the policy’s impact, which is statistically robust and significant. The lack of significant pre-trends supports the parallel trends assumption, thereby validating the DID methodology used in this study. However, it is important to acknowledge that short-term economic fluctuations or localized shocks may have influenced the cities differently before the policy was enacted, although these factors do not undermine the robustness of the policy’s overall effects.

Fig 1 illustrates the regression coefficients for the common trend test before and after the implementation of the rent-buy policy, offering a visual evaluation of the parallel trends assumption. The blue and green lines represent the regression coefficients for first-hand and second-hand house prices, respectively. Before policy implementation, the coefficients are close to zero and show no significant deviations, suggesting that the treated and control cities had similar house price trends prior to the policy. This supports the parallel trends assumption, which is critical for the validity of the Difference-in-Differences (DID) estimation employed in this study.

After the policy implementation, both lines exhibit a clear downward trend, with coefficients becoming significantly negative. This result aligns with the findings in Table 3, where the policy effect is estimated as −0.051 for first-hand house prices and −0.071 for second-hand house prices, both statistically significant at the 1% level. The observed downward trend after the policy implementation indicates that the rent-buy policy effectively reduced house price growth over time.

In conclusion, the regression coefficients before and after the policy implementation provide robust support for the validity of the DID model. The lack of significant pre-trends reinforces the parallel trends assumption, enabling reliable estimation of policy effects. While the post-policy coefficients are consistent with Table 3, it is crucial to acknowledge potential limitations such as short-term economic fluctuations or localized shocks that may have differentially influenced the treated and control cities prior to the policy. These factors should be considered when interpreting the results, despite the robustness of the overall policy effects.

### 5.3 Rent-buy policy and migration patterns

To further understand the impact of the rent-buy policy, this study also examines its effects on population migration, as housing affordability is often closely linked to migration decisions. The implementation of the rent-buy policy may influence population dynamics by making rental housing more accessible, thereby affecting individuals’ decisions to move into or out of treated cities.

Migration data were collected from city-level population statistics, including net migration rates, migration inflows, and outflows, both before and after the policy implementation. These data allow us to observe the population changes in both experimental and control cities, providing insights into how the rent-buy policy has affected migration patterns.

Migration rates serve as an important supplementary indicator of the policy’s impact, as they reflect changes in the attractiveness of cities for potential residents. By making rental housing more affordable and improving living conditions, the rent-buy policy may lead to increased migration inflows into treated cities, especially for younger individuals seeking employment opportunities. Conversely, better rental conditions may also reduce out-migration, as residents are more likely to remain if they can find affordable housing locally.

**Fig 2:**
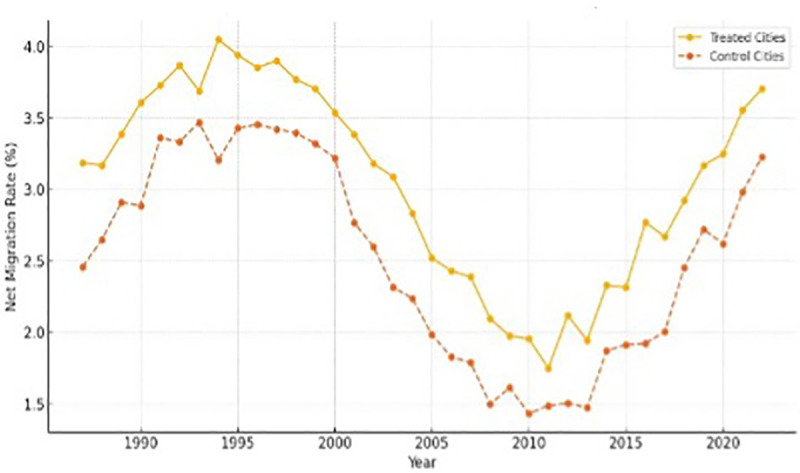
Trends in net migration rates.

Fig 2 shows the trends in net migration rates for the treatment and control cities before and after the implementation of the rent-buy policy. The results suggest that treated cities experienced a relative increase in net migration compared to control cities, indicating that the policy may have enhanced the attractiveness of these cities for potential migrants.

### 5.4 Robustness test

#### 5.4.1 PSM-DID robustness test.

To demonstrate that the rent-buy policy is robust to house prices, this section re-estimates the sample using the PSM-DID model. This is done by regressing the policy dummy variables with the control variables as explanatory variables and selecting matches from the control group whose propensity scores are closest to those of the treatment group to minimize estimation bias. By analyzing the results of the balanced trend hypothesis test the significance test level of the matched variables did not pass the 5% test value, indicating that the matched data are not significantly different. In this paper, three different matching algorithms were used to perform DID regression on each of them and the results are shown in Table 4 Models 1–3.

**Table 4:** Robustness tests of rent-buy policies on house prices.

Variables	Model1 Radius matching	Model 2 Proximity matching	Model 3 Kernel matching
Treat*policy	−0.022*** (0.008)	−0.022*** 0.008)	−0.021*** (0.008)
Cons	0.418*** (0.139)	0.421*** (0.144)	0.420*** (0.143)
R^2^	0.440	0.441	0.439
Time	YES	YES	YES
City	YES	YES	YES
Control variables	YES	YES	YES
Obs	6975	6975	6975

*, **, and *** indicate statistically significant at the 10%, 5%, and 1% levels, respectively, and robust standard errors are reported in parentheses.

According to the regression results shown in Table 4 Models 1–3, Rent-Buy policy has negative influence on house price regardless of whether radius matching, proximity matching or sum matching is used. This result confirms that the benchmark regression results are robust.

#### 5.4.2 Change policy variable and extended controls.

To further verify the robustness of the above findings, we replace the dynamic house price with a static house price index as the explanatory variable. Although the static index does not explicitly incorporate the cultural characteristics of Chinese households, it effectively captures the rate of house price changes, facilitating an examination of price fluctuations across different periods.

In Columns (3) and (4) of Table 5, we introduce additional controls, including suburban housing supply, land prices, land supply area, and the proportion of young population. These variables represent market and demographic factors that may shape average housing prices beyond the policy intervention itself.

**Table 5:** Change policy variable.

Variables	First hands house price	Second hands house price	First hands house price	Second hands house price
Treat*policy	−0.021*** (0.002)	−0.011*** (0.003)	−0.020*** (0.002)	−0.010*** (0.003)
Control variables	Y	Y	Y	Y
Suburban Housing Supply			−0.009 (0.006)	−0.006 (0.007)
Land Price Index			0.018 (0.010)	0.027** (0.012)
Land Supply (Area)			−0.005 (0.004)	−0.007 (0.005)
Young Population Share			0.003 (0.001)	0.002 (0.001)
Fix effects	Y	Y	Y	Y
Time effects	Y	Y	Y	Y
R	0.317	0.272	0.039	0.027
Obs	6975	6975	6975	6975

*, **, and *** indicate statistically significant at the 10%, 5%, and 1% levels, respectively, and robust standard errors are reported in parentheses.

The results demonstrate that while these additional controls generally behave as expected—for instance, an increase in suburban housing supply slightly reduces central city house prices—the Treat×Policy coefficient remains negative and statistically significant. Specifically, for first-hand house prices (Column 3), the coefficient changes only marginally from −0.021 to −0.020, and for second-hand house prices (Column 4), from −0.011 to −0.010. This consistency underscores that the rent-buy policy continues to exert a dampening effect on house price growth, aligning with our central argument that it improves house price affordability.

Although incorporating these market and demographic variables provides additional explanatory power, it does not weaken or overturn the policy effect. Instead, it confirms that while multiple factors contribute to housing price variations, the rent-buy policy remains a critical determinant with a robust negative impact on house price appreciation.

## 6 Further discussion

Through the double difference model empirical test and theoretical analysis above, this paper finds that the rent-and-buy policy indeed significantly suppresses the rise of new housing prices and second-hand housing prices, and this result also passes a series of robustness tests. However, over the past year since the implementation of the rent-and-buy policy in the pilot cities, which types of housing have been mainly affected? And what kind of impact has it had on housing rents respectively? In the following, we analyze the effect of the implementation of the rent-purchase policy on housing of different sizes and changes in rents, to verify the research hypothesis 2.

In the following, we further evaluate the policy effects of the rent-purchase policy on housing of different floor areas (considering the availability of data, this part adopts the second dynamic house price index as the explanatory variable), and subdivide the newly-built houses and second-hand houses into three types of housing of90 ~ 144 square meters, and more than 144 square meters respectively, and the results of the double-difference regression are shown in Table 6. Table 6 shows that: first, the coefficients of the interaction term ${\text{ Treat }}_{it}\times {\text{ Policy }}_{it}$are all significantly negative, indicating that the rent-and-buy policy has a significant inhibitory effect on the prices of housing of different floor areas, which is consistent with the findings of the previous study; second, the interaction terms ${\text{ Treat }}_{it}\times {\text{ Policy }}_{it}$of columns (1), (3) reflecting the effects of the policy on newly-built housing, and columns (2), (4) reflecting the effects of the policy on second-hand housing, have significant negative coefficients. The absolute values of the coefficients of the interaction term ${\text{ Treat }}_{it}\times {\text{ Policy }}_{it}$all show a decreasing trend, indicating that the inhibitory effect of rent-and-buy on house prices decreases with the increase of floor area, that is to say, rent-and-buy policy has a greater impact on the price of small-sized housing; third, comparing the magnitude of the coefficients of the interaction term ${\text{ Treat }}_{it}\times {\text{ Policy }}_{it}$of the newly-built and second-hand houses with the same floor area, it is found that Third, comparing the size of the coefficient of the interaction term ${\text{ Treat }}_{it}\times {\text{ Policy }}_{it}$for new houses and second-hand houses with the same floor area, it is found that the absolute values of the coefficients of the interaction terms of new houses are larger than the absolute values of the coefficients of the interaction terms of second-hand houses, which means that the rent-purchase policy has a greater impact on the prices of newly built houses than the prices of second-hand houses.

**Table 6:** Mechanism discussion of difference area.

Variables	Group 1(90–144)		Group 2(>=144)	
	First hands house price (1)	Second hands house price (2)	First hands house price (3)	Second hands house price (4)
Treat*policy	−0.217*** (0.021)	−0.171 (0.172)	−0.154*** (0.019)	−0.021*** (0.001)
Control variables	Y	Y	Y	Y
Fix effects	Y	Y	Y	Y
Time effects	Y	Y	Y	Y
R	0.421	0.412	0.387	0.397
Obs	4710	2265	3787	3188

*, **, and *** indicate statistically significant at the 10%, 5%, and 1% levels, respectively, and robust standard errors are reported in parentheses.

## 7 Conclusions and suggestions

### 7.1 Conclusions

In this study, we employ the discounted cash flow methodology and integrate demographic and consumption variables into the conceptual framework of the intergenerational overlap model to devise a real estate bubble index tailored to China’s specific context.

Our findings reveal the emergence of significant housing price affordability not only in Tier 1 cities but also across a broad spectrum of Tier 2, Tier 3, and even Tier 4 cities, with certain Tier 5 cities also demonstrating varying degrees of bubble intensity. Notably, in specific Tier 3 and Tier 4 cities, the bubble extent surpasses that of Tier 1 cities. Nevertheless, despite fluctuations, there is no discernible real estate bubble in the national average price over the past decade, underscoring the necessity of localized analysis to uncover the nuanced landscape.

Moreover, this paper delves into the impact of the rent-purchase policy on housing prices across 279 cities from a macro policy perspective, elucidating its influence on housing market development. The study results elucidate that: (1) the rent-purchase policy significantly curbs house price escalation, with greater impact observed on new house prices compared to second-hand house prices, indicating the policy’s facilitation of the transition from an incremental housing market to a stock market; (2) the effect of the policy is more pronounced for smaller floor areas, suggesting heightened sensitivity among consumers purchasing smaller units. This underscores the rent-purchase policy’s ability to address housing demand within the low-income demographic. Simultaneously, it underscores the significance of cultivating and expanding the housing rental market as a pivotal means of meeting the housing needs of these groups.

From a Confucian cultural perspective, these findings reflect the broader societal values of balance, fairness, and familial stability. Confucianism emphasizes the importance of social harmony, and policies that aim to reduce housing affordability issues align with these ideals. The rent-purchase policy, by mitigating housing price escalation, contributes to social equity by ensuring that families, particularly those from lower income groups, have access to affordable housing. This reflects Confucian values of social welfare and collective well-being, which prioritize family and community stability over individual economic gain.

Furthermore, Confucianism’s emphasis on long-term societal planning resonates with the macroeconomic policies examined in this study. The cultivation of a rental housing market to address affordability echoes the Confucian belief in providing for the long-term welfare of society. Such policies help to create a more equitable distribution of resources, consistent with Confucian principles of fairness and moral responsibility.

These conclusions not only contribute to our understanding of real estate bubbles and affordability in China but also demonstrate how Confucian cultural values can play a critical role in shaping the success of economic and housing policies. The integration of cultural perspectives into policy design could offer deeper insights into policy impacts and effectiveness, thereby contributing to more sustainable and socially responsible housing solutions.

### 7.2 Policy implications

China’s real estate market is at a critical crossroads. The rapid decline in house prices poses a significant threat to property developers’ profitability, potentially leading to losses and defaults fueled by high leverage and heavy reliance on bank financing. Such a scenario could escalate non-performing loans, potentially triggering bank failures and systemic financial crises.

Compounding this challenge is the government’s reliance on land sale revenues, which further complicates the situation. A substantial decrease in land prices would significantly reduce government income, exacerbating the management of substantial public debt. Consequently, local governments have a vested interest in maintaining high property prices.

To navigate these complexities, we propose a dual-focused strategy aimed at mitigating the current bubble’s economic repercussions and curbing future bubble expansion.

To alleviate the impact of the existing bubble, the government should implement supportive fiscal measures such as tax breaks and monetary policies like reducing residential mortgage rates. These measures could bolster the economy’s inherent ability to absorb affordability.

Preventing further bubble growth necessitates stringent controls on real estate speculation and reducing local governments’ financial dependence on land sales. Additional strategic measures include increasing down payment requirements for property purchases, elevating mortgage rates for secondary homes, reducing standard housing sizes, and introducing property taxes for individuals with multiple properties. These measures collectively aim to stabilize the real estate market, mitigate risks, and foster sustainable economic growth.

### 7.3 Limitations and future research

Despite these insights, our study has certain limitations.

**Data Constraints**: Our city-level bubble index may underestimate or overlook micro-level nuances, especially in rapidly changing local markets. Incorporating more granular data (e.g., neighborhood- or household-level) could refine the results.

**Policy Scope**: We focus on the rent-purchase framework; however, China’s real estate market is influenced by a host of concurrent regulations (such as purchase restrictions or mortgage caps). Future research could disentangle these overlapping effects to gauge each policy’s independent impact.

**Cultural and Behavioral Factors**: Confucian norms and familial support for housing play a strong role in shaping market behavior, yet quantifying such cultural influences remains challenging. Further studies with micro-level surveys or qualitative approaches could yield deeper insights into these social dynamics.

### 7.4 Specific implementation path

Building on these findings, we propose a step-by-step implementation path for local authorities and stakeholders. Pilot Testing: Expand rent-purchase schemes initially in cities showing early signs of bubble escalation, coupled with robust data collection to monitor effectiveness.Gradual Rollout of Property Taxes: Phase in property taxes on second or multiple-home ownership, giving local governments time to develop alternative revenue sources (e.g., local consumption or service taxes). Data Integration and Transparency: Enhance information-sharing platforms among municipalities, banks, and developers to ensure accurate tracking of land supply, housing transactions, and affordability metrics.Incentives for Smaller Units and Rental Supply: Encourage developers to build smaller, more affordable units through subsidies or tax incentives, while simultaneously protecting tenant rights to foster a culture of renting.

By combining immediate fiscal measures with forward-looking regulatory actions, policymakers can mitigate imminent financial risks and guide the market toward long-term stability. These recommendations integrate both economic and socio-cultural considerations, recognizing the distinct nature of China’s real estate environment and the critical importance of housing affordability for social harmony and economic resilience.
